# Community-acquired pneumonia: economics of inpatient medical care vis-à-vis clinical severity*,**

**DOI:** 10.1590/S1806-37132015000100007

**Published:** 2015

**Authors:** Vojislav Cupurdija, Zorica Lazic, Marina Petrovic, Slavica Mojsilovic, Ivan Cekerevac, Nemanja Rancic, Mihajlo Jakovljevic

**Affiliations:** University of Kragujevac, Center for Clinical Medicine, Pulmonology Department, Kragujevac, Serbia. Pulmonology Department, University of Kragujevac Center for Clinical Medicine, Kragujevac, Serbia; University of Kragujevac, Center for Clinical Medicine, Pulmonology Department, Kragujevac, Serbia. Faculty of Medical Sciences, University of Kragujevac, Kragujevac, Serbia; and Head. Pulmonology Department, University of Kragujevac Center for Clinical Medicine, Kragujevac, Serbia; University of Kragujevac, Center for Clinical Medicine, Pulmonology Department, Kragujevac, Serbia. Faculty of Medical Sciences, University of Kragujevac, Kragujevac, Serbia; and Internist. Pulmonology Department, University of Kragujevac Center for Clinical Medicine, Kragujevac, Serbia; University of Kragujevac, Center for Clinical Medicine, Pulmonology Department. Pulmonology Department, University of Kragujevac Center for Clinical Medicine, Kragujevac, Serbia; University of Kragujevac, Center for Clinical Medicine, Kragujevac, Serbia. Intensive Care Unit, University of Kragujevac Center for Clinical Medicine, Kragujevac, Serbia; University of Defence, Military Medical Academy, Medical Faculty, Belgrade, Serbia. Centre for Clinical Pharmacology, Medical Faculty, Military Medical Academy, University of Defence, Belgrade, Serbia; University of Kragujevac, Faculty of Medical Sciences, Kragujevac, Serbia. Graduate Program in Health Economics and Pharmacoeconomics, Faculty of Medical Sciences, University of Kragujevac, Kragujevac, Serbia

**Keywords:** Pneumonia, Cost of illness, Costs and cost analysis, Health care costs, Hospitalization, Severity of illness index

## Abstract

**Objective:**

To assess the direct and indirect costs of diagnosing and treating community-acquired pneumonia (CAP), correlating those costs with CAP severity at diagnosis and identifying the major cost drivers.

**Methods:**

This was a prospective cost analysis study using bottom-up costing. Clinical severity and mortality risk were assessed with the pneumonia severity index (PSI) and the mental Confusion-Urea-Respiratory rate-Blood pressure-age ≥ 65 years (CURB-65) scale, respectively. The sample comprised 95 inpatients hospitalized for newly diagnosed CAP. The analysis was run from a societal perspective with a time horizon of one year.

**Results:**

Expressed as mean ± standard deviation, in Euros, the direct and indirect medical costs per CAP patient were 696 ± 531 and 410 ± 283, respectively, the total per-patient cost therefore being 1,106 ± 657. The combined budget impact of our patient cohort, in Euros, was 105,087 (66,109 and 38,979 in direct and indirect costs, respectively). The major cost drivers, in descending order, were the opportunity cost (lost productivity); diagnosis and treatment of comorbidities; and administration of medications, oxygen, and blood derivatives. The CURB-65 and PSI scores both correlated with the indirect costs of CAP treatment. The PSI score correlated positively with the overall frequency of use of health care services. Neither score showed any clear relationship with the direct costs of CAP treatment.

**Conclusions:**

Clinical severity at admission appears to be unrelated to the costs of CAP treatment. This is mostly attributable to unwarranted hospital admission (or unnecessarily long hospital stays) in cases of mild pneumonia, as well as to over-prescription of antibiotics. Authorities should strive to improve adherence to guidelines and promote cost-effective prescribing practices among physicians in southeastern Europe.

## Abstract (pt)

**Objetivo:**

Avaliar os custos médicos diretos e indiretos de diagnóstico e tratamento para pacientes com pneumonia adquirida na comunidade (PAC), correlacionando-os com a gravidade da PAC ao diagnóstico e identificando os principais fatores de custo.

**Métodos:**

Análise de custos prospectiva utilizando custo *bottom-up*. A gravidade clínica e o risco de mortalidade foram determinados através de *pneumonia severity index* (PSI) e a escala *mental* **C** *onfusion-*U*rea-*R*espiratory rate-*B*lood pressure-age ≥ 65 years* (CURB-65), respectivamente. A amostra foi composta por 95 pacientes hospitalizados devido a PAC recém-diagnosticada. A análise foi realizada em uma perspectiva social com um horizonte de tempo de um ano.

**Resultados:**

Expressos em média ± desvio-padrão em euros, os custos médicos diretos e indiretos por paciente com PAC foram de 696 ± 531 e 410 ± 283, respectivamente, sendo, portanto, o custo total por paciente de 1.106 ± 657. O impacto orçamentário combinado deste grupo de pacientes em euros foi de 105.087 (66.109 e 38.979 nos custos diretos e indiretos, respectivamente). Os principais fatores de custo, em ordem descendente, foram custo de oportunidade (perda de produtividade); diagnóstico e tratamento de comorbidades; e administração de medicamentos, oxigênio e derivados do sangue. Os escores CURB-65 e PSI correlacionaram-se com os custos indiretos do tratamento da PAC. O escore PSI correlacionou-se positivamente com a frequência global no uso de serviços médicos. Nenhum dos escores mostrou uma relação clara com os custos diretos do tratamento da PAC.

**Conclusões:**

A gravidade clínica na admissão parece não se correlacionar com os custos do tratamento da PAC. Esses custos são principalmente causados por internações hospitalares desnecessárias (ou por internação desnecessariamente prolongada) em casos de pneumonia leve, assim como pela prescrição exagerada de antibióticos. As autoridades devem se esforçar para melhorar a adesão às diretrizes e promover práticas de prescrição custo-efetivas entre os médicos do sudeste da Europa.

## Introduction

Community-acquired pneumonia (CAP) remains an ongoing challenge for health care facilities specializing in pulmonary medicine and critical care, across the globe. ^(^ 1 ^)^ Its treatment is still demanding, and outcomes remain unpredictable, despite the unprecedented innovation in the development of new antibiotics. ^(^ 2 ^)^ One major cause of the difficulties experienced in and the frequently unsatisfactory clinical outcomes of the treatment of CAP is poor adherence to clinical practice guidelines, as demonstrated in a recent study conducted in Brazil.^(^ 3 ^)^ In the context of increased expectations that health care systems will deliver cost-effective care, the financial burden of CAP has attracted the attention of experts. ^(^ 4 ^)^ Various measures have been proposed and implemented to contain the costs related to the care of patients with CAP while preserving satisfactory clinical outcomes. ^(^ 5 ^)^ Among the most successful of such measures is prioritizing outpatient diagnosis and treatment over hospital admission. Commonly reported results of this strategy are lower frequency of hospital infections with multidrug-resistant bacteria and fewer resources spent on the diagnosis and treatment of associated comorbidities.^(^ 6 ^)^

In comparison with other countries in the Western Balkans, one peculiarity of Serbia, which is the largest health care market in the region, is that its health care system is rather typical of the broader Eastern European context. ^(^ 7 ^)^ Health expenditures continue to grow faster than the gross domestic product available among the high- and upper middle-income economies of the region. ^(^ 8 ^)^ The population continues to age while incidence and prevalence rates of the leading diseases common to the upper socioeconomic classes are still rising. ^(^ 9 ^)^ Local data regarding the financial burden of CAP remain scarce across the region.^(^ 10 ^)^ Many policy makers have begun to realize that a more robust evidence base is needed in order to make informed decisions on resource allocation. In light of current weaknesses of regional health financing, funding the quest for knowledge of the local cost drivers of key clinical conditions represents a valuable investment in the future of emerging markets. ^(^ 11 ^)^

## Methods

This was a prospective non-interventional clinical study with retrospective insight into pneumonia-related resource use and the direct costs of medical care, as well as indirect costs associated with absenteeism and the related productivity losses. Our aim was to assess the direct and indirect costs of diagnosis and medical treatment of patients with CAP, as well as to determine whether those costs correlate with CAP severity (stage) and clinical status at diagnosis. The analysis was run from a societal perspective with a time horizon of one year, and we used bottom-up costing.

Patients were recruited from among inpatients recently diagnosed with CAP and admitted to the University of Kragujevac Center for Clinical Medicine, in Kragujevac, Serbia. The Center, which is one of several large tertiary care facilities in the country, with 1,300 beds, provides specialty care for central and western Serbia. Common clinical practice at the facility is mostly in line with current guidelines for the diagnosis and treatment of respiratory infections. ^(^ 12 ^)^

We included patients ≥ 18 years of age who had received a diagnosis of CAP, confirmed by laboratory tests and imaging. A diagnosis of CAP was defined by signs of new infiltration on chest X-rays, together with at least one of the following symptoms: newly occurring cough; abnormal body temperature (< 35.6ºC or > 37.5ºC); and an abnormal blood cell count, defined as leukocytosis with a "left shift" or neutropenia. Patients who had a recent prior hospital admission (within the last 15 days) for any reason were excluded, as were those presenting with clinical signs indicative of pulmonary tuberculosis and those with severe pneumonia, requiring urgent admission to the intensive care unit and assisted ventilation.

Patients were enrolled consecutively over a period of 16 months. They were assessed by the attending physicians (internists who were subspecialists in pulmonology). The majority of the eligible patients (those meeting the study criteria) were recruited. Annually, there are approximately 200 hospital admissions for CAP, as well as up to 500 cases of CAP treated on an outpatient basis, at the target facility. Approximately 30% of the inpatients initially included were lost to follow up, due to loss of contact; a change of permanent residence and contact data; or voluntary withdrawal from the study.

At admission, the clinical evolution and severity of the infection were assessed with the pneumonia severity index (PSI), ^(^ 13 ^)^ as well as the score on the scale known as CURB-65, an acronym based on the key terms of each risk factor evaluated (i.e., mental Confusion, Urea, Respiratory rate, Blood pressure, and age ≥ 65 years), which is used in order to quantify mortality risk. ^(^ 14 ^)^ Both of these scales are physician-oriented and have been widely adopted as reliable and valid clinical instruments. They are used worldwide to inform decisions regarding treatment, as well as to evaluate the likely prognosis.

Follow-up and observation took place from September of 2012 through December of 2013. The patient sample was representative of the population of central Serbia. Clinical background data were obtained by the attending physicians during prospective clinical follow-up. The results of a variety of laboratory tests and other diagnostic measures, as well as physical examination findings, are provided for the first and last day of hospitalization. We also determined 30-day mortality after discharge. 

Patterns of resource use-frequency of physical examinations, bronchoscopies, sputum cultures, blood cultures, chest X-rays, and blood tests, as well as drug doses, etc.-were prospectively reported by the attending physicians and nurses. Direct medical costs were calculated on the basis of data available in the University of Kragujevac Center for Clinical Medicine electronic database of discharge invoices. The database contained exact prices of goods and services consumed in relation to the diagnosis and treatment of CAP. These prices were registered, at the time that the services were provided, by the primary payer, the National Health Insurance Fund of the Republic of Serbia. ^(^ 15 ^)^ We calculated indirect costs using Grossman's human capital approach. ^(^ 16 ^)^ Pneumonia caused absenteeism and resulted in opportunity costs (lost productivity), as calculated on the basis of the length of hospital stay; employment status of the patient and caregiver; and history of home care. We monetized lost work time on the basis of monthly salaries by occupation, according to the current official average values published by the Republic of Serbia. ^(^ 17 ^)^

All statistical analyses were performed with the SPSS Statistics software package, version 19.0 (IBM Corporation, Armonk, NY, USA). Continuous variables are presented as mean ± standard deviation, with ranges or with 95% confidence intervals. Categorical variables are presented as proportions of the sample as a whole. To test the significance of differences between pre- and post-treatment values for continuous variables, we used Student's t-tests for dependent samples or the Wilcoxon signed-rank test, depending on the normality of the data distribution (Kolmogorov-Smirnov test results). The significance of differences between two independent samples for continuous variables was measured with Student's t-tests or the Mann-Whitney U test, also depending on the normality of the data distribution. For comparisons of continuous variables among three or more groups, we used one-way ANOVA or the Kruskal-Wallis test, again depending on the normality of the data distribution. Linear correlations between key variables were tested by Spearman's and Pearson's correlation coefficients. The strength of each correlation was assessed according to Cohen's kappa (≥ 0.5 = extremely strong; 0.3-0.49 = moderate; and ≤ 0.29 = weak). Predictors of fatal outcome were subjected to multiple logistic regression, and the results are expressed as odds ratios. The level of statistical significance was set at p < 0.05.

Patient data were handled in accordance with the Declaration of Helsinki and with Serbian national legislation on biomedical research in human subjects. The study was approved by the Research Ethics Committee of the University of Kragujevac Center for Clinical Medicine (Protocol no. 01-2349). 

## Results

The study sample was well-balanced in terms of the gender distribution, males and females respectively accounting for 46 (48.5%) and 49 (51.5%) of the 95 patients evaluated. The mean age was 63.46 ± 14.83 years (range, 23-92 years), retired individuals comprising 52.6% of the sample. The most significant risk factors were smoking and low rates of vaccination (against influenza and *Streptococcus pneumoniae*). In the vast majority of the cases, the onset of the clinical symptoms of CAP occurred at least 7 days before hospital admission (Table 1). At admission, 11 (11.6%) of the 95 patients exhibited a high degree of clinical severity (advanced stage), with impaired consciousness. The majority of patients had at least one significant comorbidity disorder, the most common comorbid condition being essential hypertension. According to the PSI scores at admission, there was a clear predominance of class III (score range, 71-90) and class IV (score range, 91-130). On the basis of the CURB-65 scores at admission, 58 (61%) of the patients were in the low-risk group and therefore might have been candidates for outpatient treatment. Nevertheless, all of the patients in our sample were admitted to the hospital. The mean hospital stay was two weeks. Thereafter, 52 (54.7%) of the 95 patients received home care, and the mean length of follow-up treatment was approximately ten days. In most cases, home care was provided by a member of the immediate or extended family. Although most of the patients recovered fully after hospital discharge, five died during the follow-up period. On the basis of the multiple logistic regression analysis we concluded that a single determinant, the CURB-65 score, is a significant predictor of mortality (OR =12,60). All of the patients who died had been classified as being in the CURB-65 moderate-risk or severe-risk group.

**Table 1 -:** Baseline demographic characteristics, lifestyle risk factors, clinical severity, and mortality risk in a sample of patients hospitalized with community-acquired pneumonia in Serbia.

Variable	(N = 95)^a^
Age	63.46 ± 14.83 (23-92)
Body mass index (kg/m^2^)	24.58 ± 5.36 (13.84-41.40)
Occupation	
Unknown, n (%)	19 (20)
Unemployed, n (%)	7 (7.4)
Homemaker, n (%)	3 (3.2)
Retired, n (%)	50 (52.6)
Unskilled laborer, n (%)	11 (11.6)
Health care worker, n (%)	1 (1.1)
Farmer, n (%)	1 (1.1)
Self-employed, n (%)	3 (3.2)
Nursing home occupant, n (%)	2 (2.1)
Current smoker, n (%)	44 (46.3)
Smoking history (pack-years)	
All patients	18.32 ± 22.85 (0-100)
Smokers only	37.05 ± 19.49 (0.4-100)
Previous vaccination against influenza	1 (1.1)
Previous vaccination against Streptococcus pneumoniae	1 (1.1)
Previous hospitalization for pneumonia, n (%)	14 (14.7)
History of alcohol consumption, n (%)	10 (10.5)
Pneumonia severity index	
Total score	85.08 ± 33.95 (13-182)
Class I (≤ 50), n (%)	15 (15.8)
Class II (51-70), n (%)	18 (18.9)
Class III (71-90), n (%)	22 (23.2)
Class IV (91-130), n (%)	32 (33.7)
Class V (> 131-395), n (%)	8 (8.4)
CURB-65 mortality risk (score)	
Low (0-1), n (%)	58 (61.1)
Moderate (2), n (%)	29 (30.5)
Severe (3-5), n (%)	8 (8.4)
Length of hospital stay (days)	14.62 ± 7.081 (3-48)
Pre-admission duration of clinical signs and symptoms of CAP (days)	6.44 ± 5.60 (1-30)
Medical care provided by a family member, n (%)	52 (54.7)
Survival at 30 days after discharge	
Yes, n (%)	90 (94.7)
No, n (%)	5 (5.3)

CURB-65: (scale based on) mental Confusion-Urea-Respiratory rate-Blood pressure-age ≥ 65 years; and CAP: communityacquired pneumonia.

a Results presented as mean ± SD (range) except where otherwise indicated.

Most of initial laboratory and blood test results were far more concerning than were those obtained at discharge, indicating successful recovery. Clinical complications of pulmonary infection were quite common. The most common such complications were (in descending order) respiratory insufficiency, PaO_2_ < 60 mmHg, anemia, pleural effusion, and diarrhea (Table 2).

**Table 2 -:** Clinical parameters, symptoms, comorbidities, and clinical complications in a sample of patients hospitalized with community-acquired pneumonia in Serbia.

Category	Variable	(N = 95)
Clinical presentation	Body temperature (°C), mean ± SD (range)	38.19 ± 0.94 (36-40)
	Respiratory rate (breaths/min), mean ± SD (range)	19.61 ± 4.73 (12-36)
	Heart rate (bpm), mean ± SD (range)	94.45 ± 18.58 (55-150)
	Systolic blood pressure (mmHg), mean ± SD (range)	124.37 ± 19.16 (75-170)
	Diastolic blood pressure (mmHg), mean ± SD (range)	75.05 ± 10.30 (55-110)
	Cough, n (%)	80 (84.2)
	Productive cough, n (%)	49 (51.6)
	Dyspnea, n (%)	55 (57.9)
	Chest pain, n (%)	44 (46.3)
	Impaired consciousness, n (%)	11 (11.6)
	ICU admission, n (%)	9 (9.5)
	Artificial ventilation (assisted breathing), n (%)	0 (0)
Comorbid disorders	Coronary heart disease, n (%)	17 (17.9)
	Heart failure, n (%)	21 (22.1)
	Heart valves damage, n (%)	4 (4.2)
	Essential hypertension, n (%)	58(61.1)
	Asthma, n (%)	10 (10.5)
	COPD, n (%)	21 (22.1)
	Kidney failure, n (%)	11 (11.6)
	Liver failure, n (%)	2 (2.1)
	Encephalopathy, n (%)	4 (4.2)
	Diabetes mellitus, n (%)	29 (30.5)
	Cancer (any malignancy), n (%)	6 (6.3)
Clinical complications	Respiratory failure, n (%)	39 (41.1)
	PaO_2_ < 60 mmHg, n (%)	32 (33.7)
	Pleural effusion, n (%)	23 (24.2)
	Pulmonary embolism, n (%)	1 (1.1)
	Pneumothorax, n (%)	0 (0)
	Heart failure, n (%)	17 (17.9)
	Gastrointestinal bleeding, n (%)	1 (1.1)
	Diarrhea, n (%)	21 (22.1)
	Hemoptysis, n (%)	9 (9.5)
	Empyema, n (%)	1 (1.1)
	Leukopenia, n (%)	2 (2.1)
	Anemia, n (%)	28 (29.5)
	Platelet deficiency, n (%)	2 (2.1)
	Stroke, n (%)	2 (2.1)
	Lung abscess, n (%)	3 (3.2)

With regards to the economics of inpatient treatment for CAP, the diagnostic tests most frequently ordered by the attending physicians at the facility under study were chest X-ray, electrocardiography, laboratory analyses (hematology and biochemistry), arterial blood gas analysis, and spirometry (see Table 3). The medications most often prescribed for the treatment of CAP, according to the mean number of defined daily doses (DDDs) per patient, were as follows: levofloxacin (8.57 DDDs); methylprednisolone (5.04 DDDs); aminophylline (4.73 DDDs); ceftriaxone (3.54 DDDs); fluticasone+salmeterol (0.73+2.20 DDDs); ceftazidime (2.47 DDDs); amikacin (2.03 DDDs); fenoterol+ipratropium bromide (0.76+1.60 DDDs); ertapenem (1.83 DDDs); and acetylcysteine (1.81 DDDs). Antibiotics were the strongest single contributor to the acquisition costs of medications.

**Table 3 -:** Patterns of resource use and frequency of pneumonia-related clinical interventions in a sample of patients hospitalized with community-acquired pneumonia in Serbia.

Intervention	Per-patient frequency of use		Total number of examinations	Patients undergoing the examinations
	Mean ± SD	(range)		n (%)
Electrocardiography	2.92 ± 2.56	0-14	277	94 (98.9)
Bronchoscopy	0.18 ± 0.39	0-1	17	17 (17.9)
Thoracocentesis	0.09 ± 0.39	0-2	9	6 (6.4)
Thoracic drainage	0.01 ± 0.10	0-1	1	1 (1.1)
Spirometry	1.12 ± 1.47	0-7	106	52 (54.6)
Chest CT	0.27 ± 0.45	0-1	26	26 (27.4)
Chest X-ray	4.08 ± 2.42	0-20	388	95 (100)
Blood culture	0.26 ± 0.64	0-2	25	15 (15.8)
Sputum culture	0.94 ± 1.45	0-6	89	40 (42.1)
Blood workup	2.35 ± 1.16	0-8	223	93 (97.9)
Serum biochemistry	2.34 ± 1.23	0-8	222	92 (96.8)
Routine coagulation tests	0.46 ± 1.13	0-9	44	26 (27.4)
Viral immunoassays	0.07 ± 0.26	0-1	7	7 (7.4)
Blood-gas analysis	2.20 ± 3.64	0-19	209	54 (56.8)

Expressed as mean ± standard deviation (95% CI), the direct and indirect costs per CAP patient, in Euros, were 696 ± 531 (30 to 589) and 410 ± 283 (353 to 467), respectively, the total per to patient cost therefore being 1,106 ± 657 (974 to 1,238). The combined budget impact of our patient cohort was 105,087 (66,109 and 38,979 in direct and indirect costs, respectively). As can be seen in Table 4, the major cost drivers were as follows (values in Euros): general medical care (32 ± 32 [26 to 39]); administration of medications, oxygen, and blood derivatives (178 ± 211 [135 to 220]); laboratory tests and imaging (52 ± 53 [42 to 63]); consultations and surgical interventions (30 ± 38 [22 to 38]); administrative and supporting services (2 ± 20 [−2 to 6]); and the diagnosis and treatment of comorbidities (401 ± 304 [340 to 462]).

**Table 4 -:** Cost matrix of medical care for community-acquired pneumonia, including cost per patient and total expenditures, in a sample of patients hospitalized with community-acquired pneumonia in Serbia, 2012-2013.

Variable	Per patient cost, in Euros^a^	Total cost, in Euros
	Mean ± SD (range)	
General medical care	32 ± 32 (26 to 39)	3,086
Nursing care	12.1 ± 12.2 (9.6 to 14.5)	1,147
Consumables	20.4 ± 21.0 (16.2 to 24.6)	1,939
Medications, oxygen, blood, and blood derivatives	178 ± 211 (135 to 220)	16,894
Anti-infective medicines for systemic use	165.4 ± 199.1 (125.3 to 205.4)	15,710
Blood and blood derivatives	1.1 ± 10.5 (−1.0 to 3.2)	103
Antiseptics and disinfectants	0.3 ± 0.4 (0.3 to 0.4)	31
Cholesterol-lowering drugs (statins, fibrates, etc.) and dietary supplements (vitamins and minerals)	0.2 ± 1.0 (0.0 to 0.4)	21
Oxygen and systemic hormonal preparations (insulin, bisphosphonates, steroids, etc.)	10.8 ± 22.7 (6.3 to 15.4)	1,029
Laboratory analysis and imaging diagnostics	52 ± 53 (42 to 63)	4,989
Laboratory analysis	29.8 ± 29.8 (23.9 to 35.8)	2,836
Simple X-rays	3.4 ± 2.7 (2.9 to 4.4)	327
Nuclear medicine diagnostics	0.3 ± 1.9 (−0.1 to 0.6)	26
Tools and consumables	0.1 ± 0.5 (0.0 to 0.2)	7
Cardiovascular interventional radiology	12.3 ± 35.6 (5.2 to 19.5)	1,173
Tools and consumables	0.4 ± 4.1 (−0.4 to 1.2)	40
Contrast agents, film, etc., for radiological services	6.1 ± 8.8 (4.3 to 7.9)	581
Consultations and surgical interventions	30 ± 38 (22 to 38)	2,858
Consultations	28.7 ± 37.3 (21.3 to 36.2)	2,731
Surgical interventions	0.9 ± 3.9 (0.2 to 1.7)	90
Dialysis and psychiatric treatment	0.4 ± 1.4 (0.1 to 0.7)	37
Administrative and supporting services	2 ± 20 (−2 to 6)	192
Diagnosis and treatment of comorbidities	401 ± 304 (340 to 462)	38,092
Direct costs	696 ± 531 (30 to 589)	66,109
Indirect costs	410 ± 283 (353 to 467)	38,979
Total costs	1,106 ± 657 (974 to 1,238)	105,087

After dividing the patients into subgroups according to their CURB-65 score (mortality risk) at admission, we identified no significant cost differentials among the low-, moderate-, and severe-risk groups. Minor exceptions were oxygen and hormonal preparations, the administration of which was significantly more frequent among patients in the CURB-65 moderate- and severe-risk groups (p = 0.046 vs. the low-risk group). Paradoxically, the cost of CAP treatment was highest for the patients in the CURB-65 low-risk group. That finding can be explained by the fact that most of those cases were heavily dependent on home nursing care after early discharge, increasing the opportunity costs related to lost productivity (p = 0.002 vs. the moderate- and severe-risk groups).

There was a strong positive correlation between the PSI and CURB-65 scores (r = 0.663). We also found that the scores on the PSI and CURB-65 both correlated with the indirect costs of CAP treatment (r = −0.339 and r = −0.360, respectively). Greater CAP severity, as indicated by a higher PSI score, correlated with the use of imaging and laboratory tests (r = 0.177), as well as with the administration of medications, oxygen and blood derivatives (r = 0.257). The PSI score also correlated positively with the overall per-patient frequency of use of health care services (r = 0.354).

The overall costs of care were substantially lower for CAP non-survivors than for CAP survivors. That is primarily attributable to the shorter hospital stays among the former (p = 0.049), although the costs related to imaging, laboratory tests, physician consultations, and surgical costs were also significantly higher among the survivors (p = 0.004). Length of hospital stay exhibited strong positive correlations with direct, indirect, and total costs (r = 0.493, r = 0.307, and r = 0.531, respectively).

## Discussion

The results presented here are the fruit of an attempt to analyze resource use, costs and clinical practice patterns on CAP in southeastern Europe, ^(^ 10 ^)^ which, to our knowledge, constitutes the first such attempt. Similar data are readily available for a number of high income economies. In Switzerland, for example, the overall cost for a single episode of CAP in a child or adolescent is calculated to be 11,258 Swiss francs, or approximately 11,000 Euros. ^(^ 18 ^)^ These costs vary widely among economies. In Poland, the mean cost for outpatient treatment of CAP is only 186 zlotys, or approximately 43 Euros. ^(^ 19 ^)^ To date, there have been only a few cost-of-illness studies on respiratory disorders in Eastern Europe, and most of those have focused on COPD, confirming its huge economic burden. Such studies have shown that the costs of treatment increase in parallel with the degree of COPD severity, according to the Global Initiative for Chronic Obstructive Lung Disease clinical classification. ^(^ 20 ^)^ Intercountry comparisons of the costs of COPD treatment in Europe remain scarce, which hinders analysis of the key cost drivers and unique national health care settings. ^(^ 21 ^)^ Estimates of the annual economic burden of CAP have exceeded common expectations in some markets, ranging from 63 million New Zealand dollars (approximately 42 million Euros) in New Zealand to 440.7 million pounds (approximately 574 million Euros) in the United Kingdom. ^(^ 21 ^)^

Our finding that the length of hospital stay had a significant impact on the overall costs of CAP treatment is supported by well-documented evidence from other national settings. ^(^ 22 ^)^ The strongest single cost driver in our sample was the opportunity cost related to work absenteeism. In developed countries, such a cost matrix is common for the majority of noncommunicable diseases, although it is less common for short-term, communicable diseases. Our finding that the diagnosis and treatment of major comorbidities constituted the major cost driver could be explained by the rising incidence and financial burden of diseases common to the upper socioeconomic classes in Eastern Europe. Local evidence strongly supporting our findings can be found in other studies, also conducted in Serbia, evaluating the burdens of diabetes mellitus, COPD, addiction, fertility disorders, hepatitis, and cancer. ^(^ 23 ^-^ 27 ^)^

Our data, which were acquired in a prospective manner within a methodologically appropriate framework, show that only the PSI score was predictive of the volume of health care services consumed. The CURB-65 and PSI scores both showed a satisfactory positive predictive value for the opportunity costs related to lost productivity. Nevertheless, we failed to identify any significant correlations between either of those clinical assessments of severity and the direct costs of CAP treatment. The lack of any such correlation is likely the result of poor physician adherence to guidelines. ^(^ 28 ^)^ It seems that high prescription rates and the overuse of diagnostics measures (imaging and laboratory testing) are common in cases of CAP that are treated in the early, mild stages. Resource use and costs in more severe, advanced cases of CAP, in which the outcome is highly unpredictable, too frequently approach those seen in mild cases. It is likely that this is primarily attributable to long hospital stays, the routine administration of multiple antibiotics, and the use of the expensive parenteral medications preferred by physicians in the region. An excellent recent example of how adherence to clinical guidelines can downsize treatment expenditures and generate savings was provided in a controlled study of alcohol addiction conducted by our group. ^(^ 29 ^)^ Our findings in the present study underscore the need for health care policies establishing stricter supervision of standard clinical practice. Analysis of the current state of the art of CAP treatment in Serbia, the largest health care market in the Western Balkans, indicates that the allocation of resources is inefficient. Providing local physicians with better evidence-based guidelines on cost-effective medical interventions for pneumonia would likely improve clinical outcomes and generate savings.

The present study has some minor limitations. Conducting a prospective cost-of-illness study in parallel with a non-interventional clinical study in one large university hospital precludes a substantial increase in the sample size. A multicenter study conducted across several countries in the region might allow the knowledge of CAP treatment costs to be expanded beyond its current limits. Unfortunately, such an additional effort was not within the scope of the present study. Nevertheless, the results of our study, which we believe to be the first of its kind conducted in southeastern Europe, could lay the groundwork for improved cost-efficiency in the treatment of pneumonia in the region.

As previously mentioned, the dominant cost drivers were the treatment of comorbid disorders, clinical complications, and the opportunity cost related to lost productivity. In view of the high incidence of respiratory infections in European communities, health care authorities should strive to improve adherence to guidelines and promote cost-effective prescribing practices among physicians in the region. Adopting long-term strategies aimed at reshaping the mindset of regional hospital staff would help contain costs and improve clinical outcomes.
